# Treatment effects of recombinant human soluble thrombomodulin in patients with severe sepsis: a historical control study

**DOI:** 10.1186/cc10228

**Published:** 2011-05-11

**Authors:** Kazuma Yamakawa, Satoshi Fujimi, Tomoyoshi Mohri, Hiroki Matsuda, Yasushi Nakamori, Tomoya Hirose, Osamu Tasaki, Hiroshi Ogura, Yasuyuki Kuwagata, Toshimitsu Hamasaki, Takeshi Shimazu

**Affiliations:** 1 Department of Emergency and Critical Care, Osaka General Medical Center, 3-1-56 Bandai-Higashi, Sumiyoshi-ku, Osaka 558-8558, Japan; 2 Department of Traumatology and Acute Critical Medicine, Osaka University Graduate School of Medicine, 2-15 Yamadaoka Suita, Osaka 565-0871, Japan; 3 Department of Biomedical Statistics, Osaka University Graduate School of Medicine, 2-15 Yamadaoka Suita, Osaka 565-0871, Japan

## Abstract

**Introduction:**

Cross-talk between the coagulation system and inflammatory reactions during sepsis causes organ damage followed by multiple organ dysfunction syndrome or even death. Therefore, anticoagulant therapies have been expected to be beneficial in the treatment of severe sepsis. Recombinant human soluble thrombomodulin (rhTM) binds to thrombin to inactivate coagulation, and the thrombin-rhTM complex activates protein C to produce activated protein C. The purpose of this study was to examine the efficacy of rhTM for treating patients with sepsis-induced disseminated intravascular coagulation (DIC).

**Methods:**

This study comprised 65 patients with sepsis-induced DIC who required ventilatory management. All patients fulfilled the criteria of severe sepsis and the International Society on Thrombosis and Haemostasis criteria for overt DIC. The initial 45 patients were treated without rhTM (control group), and the following 20 consecutive patients were treated with rhTM (0.06 mg/kg/day) for six days (rhTM group). The primary outcome measure was 28-day mortality. Stepwise multivariate Cox regression analysis was used to assess which independent variables were associated with mortality. Comparisons of Sequential Organ Failure Assessment (SOFA) score on sequential days between the two groups were analyzed by repeated measures analysis of variance.

**Results:**

Cox regression analysis showed 28-day mortality to be significantly lower in the rhTM group than in the control group (adjusted hazard ratio, 0.303; 95% confidence interval, 0.106 to 0.871; *P *= 0.027). SOFA score in the rhTM group decreased significantly in comparison with that in the control group (*P *= 0.028). In the *post hoc *test, SOFA score decreased rapidly in the rhTM group compared with that in the control group on day 1 (*P *< 0.05).

**Conclusions:**

We found that rhTM administration may improve organ dysfunction in patients with sepsis-induced DIC. Further clinical investigations are necessary to evaluate the effect of rhTM on the pathophysiology of sepsis-induced DIC.

## Introduction

Cross-talk between the coagulation system and inflammatory reactions during sepsis causes organ damage followed by multiple organ dysfunction syndrome or even death [1-3]. Disseminated intravascular coagulation (DIC) is a strong predictor of mortality in patients with severe sepsis. Bakhttiari *et al*. [4] showed that in patients with DIC, 28-day mortality was 45%, whereas it was 25% in patients without DIC. Therefore, anticoagulant therapies have been expected to be beneficial for the treatment of not only septic coagulopathy but also severe sepsis. A mortality benefit was demonstrated when recombinant human activated protein C (rhAPC) was administered to humans in the Recombinant Human Activated Protein C Worldwide Evaluation in Severe Sepsis (PROWESS) trial [5]. In addition, *post hoc *analysis demonstrated that larger absolute reductions in mortality were found with incrementally higher baseline degrees of severity of illness [6,7]. Thus, the 2008 Surviving Sepsis Campaign Guidelines [8] downgraded the recommendation for rhAPC therapy, using the word "suggest" rather than "recommend." In contrast to rhAPC, administration of antithrombin (AT), another endogenous anticoagulant that successfully corrected experimental microvascular dysfunction, to patients with severe sepsis failed to reduce 28-day mortality in the KyberSept trial [9].

Thrombomodulin (TM) is a transmembrane protein on the endothelial cell surface that plays an important role in the regulation of intravascular coagulation [10]. Delvaeye *et al*. [11] reported that TM acts as a negative regulator of the complement system, which is activated in severe sepsis and which contributes to multiple organ failure and death [12]. Recombinant human soluble thrombomodulin (rhTM) binds to thrombin to inactivate coagulation, and the thrombin-rhTM complex activates protein C to produce activated protein C (APC), which, in the presence of protein S, inactivates factors VIIIa and Va, thereby inhibiting further thrombin formation. Moreover, the N-terminal lectin-like domain of rhTM is a unique structure that shows anti-inflammatory activity. It decreases the levels of high-mobility group box 1 (HMGB1) protein [13] and lipopolysaccharide [14] in the plasma in experimental endotoxemia. Thus, rhTM might be appropriate for the treatment of septic patients with reduced endothelial TM.

The novel biological agent rhTM was approved and is being used clinically for DIC treatment in Japan. The effects of rhTM on DIC were previously examined in a multicenter, randomized clinical trial [15] in Japan, and resolution of DIC was significantly better in the group treated with rhTM than in the group treated with unfractionated heparin. However, nonsignificant trends in favor of rhTM, as compared with heparin, were observed for mortality in patients with sepsis-induced DIC. The purpose of this study was to examine the efficacy of rhTM for treating patients with sepsis-induced DIC in terms of mortality and physiological/biochemical effects.

## Materials and methods

### Study population

The present study comprised 65 patients with sepsis-induced DIC. Inclusion criteria were a known or suspected infection on the basis of clinical data at study entry, two or more signs of systemic inflammation with at least the presence of sepsis-induced organ dysfunction, hematologic dysfunction (platelet count <80,000/mm^3^) and the necessity of mechanical ventilation to stabilize the patient's general condition. All patients fulfilled the criteria of the International Society on Thrombosis and Haemostasis classification for overt DIC. The exclusion criteria were as follows: fatal or life-threatening bleeding (intracranial, gastrointestinal or pulmonary bleeding); history of cerebrovascular disorder (cerebral bleeding or cerebral infarction) within 1 year; age ≤15 years; history of hypersensitivity to protein preparations or unfractionated heparin; pregnancy or breastfeeding; and fulminant hepatitis, decompensated liver cirrhosis or other serious liver disorder.

**Figure 1:**
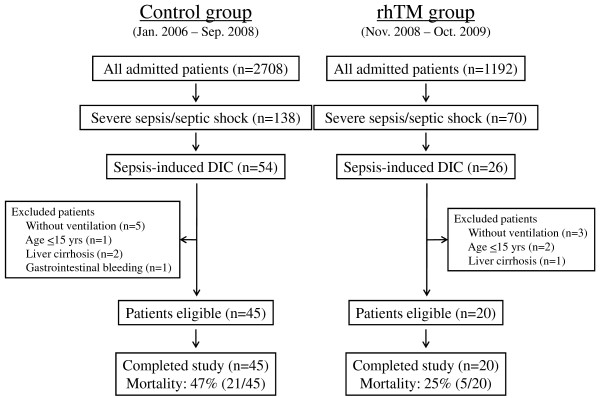
**Patient flow diagram**. rhTM, recombinant human soluble thrombomodulin; DIC, disseminated intravascular coagulation.

The patient flow diagram is shown in Figure 1. From November 2008 to October 2009, 20 patients who met the above-mentioned inclusion criteria and who were admitted to the intensive care unit (ICU) of Osaka General Medical Center, Osaka, Japan, were eligible for treatment with rhTM. Forty-five patients who met the same inclusion criteria and were admitted to the ICU from January 2006 to September 2008 were used as the comparison controls.

This study was carried out in accord with the principles of the Declaration of Helsinki. The ethics committee at our institution does not require its approval or informed consent for retrospective studies such as this study.

### Interventions

Administration of rhTM was started when the patients fulfilled the above-described inclusion criteria. rhTM treatment (0.06 mg/kg/day) was continued for six days. There was no difference in treatment strategy, equipment used or number of physicians and nurses who took care of the patients in the two periods. All patients were principally treated according to the strategy of the Surviving Sepsis Campaign Guidelines [8].

### Data collection

Patients were followed until 28 days after entry into the study. The variables considered to assess comparability among the two groups were age, sex, Acute Physiology and Chronic Health Evaluation (APACHE) II score, Sequential Organ Failure Assessment (SOFA) score, number of dysfunctional organs, site of infection and rate of positive blood culture.

We evaluated 28-day mortality and physiological and biochemical variables. Platelet counts and the levels of C-reactive protein (CRP) and fibrinogen degradation products (FDP) on sequential days were assessed. SOFA score was recorded on days 0, 1, 2, 3, 7, 14 and 28. The presence of serious adverse events related to bleeding was recorded. Serious bleeding events were defined as follows: fatal bleeding (overt bleeds considered the primary cause of death), nonfatal serious bleeding (defined as intracranial hemorrhage confirmed by brain imaging, gastrointestinal or respiratory tract bleeding uncontrollable by conservative treatments, and bleeding at a critical location such as retinal hemorrhage, major hemarthrosis or spinal hemorrhage) or any life-threatening bleeding that led to discontinuation of the administered study drug.

### Statistical analysis

Data are expressed as group means ± standard error of the mean, medians with interquartile ranges, or percentages as appropriate. Continuous variables were compared between groups by using Student's *t*-test or nonparametric test as appropriate. Categorical variables were analyzed by using the χ^2 ^test or Fisher's exact test as appropriate. Univariate analysis of time to mortality was compared by using a log-rank test. In addition, stepwise multivariate Cox regression analysis was used to assess the covariates that were associated with time to mortality. Adjusted curves of time to mortality by associated covariates were estimated.

The comparisons of SOFA scores, platelet counts and CRP and FDP levels between groups over time were analyzed by repeated measures analysis of variance (ANOVA) adjusted for the baseline values as a covariate and by *post hoc *Bonferroni test. In addition, the last-observation-carried-forward (LOCF) method [16] for missing data was used for the analysis. Missing samples occurred because of death, discharge from hospitals and samples not drawn.

A *P *value < 0.05 was considered statistically significant. Statistical analyses were performed using SPSS for Windows version 17.0 software (SPSS, Inc., Chicago, IL, USA).

## Results

### Baseline characteristics

Twenty patients were treated with rhTM (rhTM group), and 45 patients were treated without rhTM (control group). The baseline characteristics of the study population are shown in Table 1. The severity of sepsis, as indicated by APACHE II and SOFA scores, number of dysfunctional organs and rate of positive blood culture, was significantly higher in the rhTM group than in the control group (*P *< 0.05). There was no difference in source of infection between the two groups. Therapeutic interventions performed during the study are listed in Table 2. There was no significant difference in therapeutic interventions between the two groups. Because the use of rhAPC has not been approved for the treatment of severe sepsis in Japan, no patient in either group underwent rhAPC.

**Table 1:** Baseline characteristics and diagnostic data of the study population^a^

Characteristics	rhTM group (*n *= 20)	Control group (*n *= 45)	*P *value
Age, yr	67.4 ± 2.9	65.4 ± 2.7	n.s.
Male sex, %	11 (55)	25 (55)	n.s.
APACHE II score	26.5 ± 1.4	21.2 ± 0.9	0.003
SOFA score	11.5 ± 0.5	9.7 ± 0.4	0.009
Number of dysfunctional organs, *n*	3.7 ± 0.3	3.0 ± 0.2	0.04
Positive blood culture, %	11 (55)	13 (29)	0.04
Site of infection			
Lung, %	2 (10)	3 (7)	n.s.
Abdomen, %	8 (40)	25 (55)	
Urinary tract, %	6 (30)	4 (9)	
Other, %	4 (20)	13 (29)	

^a^rhTM, recombinant human soluble thrombomodulin; n.s., not significant; APACHE II, Acute Physiology and Chronic Health Evaluation II; SOFA, Sequential Organ Failure Assessment. Data are expressed as group means ± standard error of the mean or number (percent).

**Table 2:** Therapeutic interventions in the study population^a^

Interventions	rhTM group (*n *= 20)	Control group (*n *= 45)	*P *value
Mechanical ventilation, %	20 (100)	45 (100)	n.s.
Shock, %	15 (75)	32 (71)	n.s.
Use of any vasopressor, %	13 (65)	33 (73)	n.s.
Infusion within 24 hours, mL	8,600 (6,400 to 13,500)	7,600 (4,200 to 11,500)	n.s.
Antibiotics administration within 3 hours of enrollment, %	20 (100)	45 (100)	n.s.
Appropriateness of antibiotic therapy, %	16 (80)	37 (82)	n.s.
Use of low-dose steroid, %	3 (15)	14 (31)	n.s.
Renal replacement therapy, %	9 (45)	16 (36)	n.s.

^a^rhTM, recombinant human soluble thrombomodulin; n.s., not significant. Data are expressed as group medians (interquartile range) or number (percent).

### Effect of treatment on mortality

**Figure 2:**
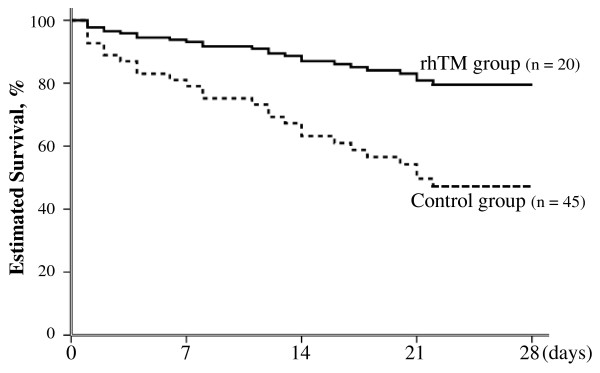
**Adjusted estimated survival curves by covariates of APACHE II score and sex in final multivariate Cox regression models**. The solid line represents patients in the rhTM group, and the dotted line represents patients in the control group. Treatment with rhTM was associated with a significantly higher rate of survival (*P *= 0.027 by stratified Cox regression analysis). APACHE II, Acute Physiology and Chronic Health Evaluation II; rhTM, recombinant human soluble thrombomodulin.

The 28-day crude mortality rate was 25% (five of twenty patients) in the rhTM group and 47% (21 of 45 patients) in the control group. There was no difference between the two groups in unadjusted mortality (*P *= 0.09 by log-rank test). Because a significant difference existed in baseline severity of illness between the two groups, we performed Cox regression analysis to adjust for these possible confounders. We assessed a total of seven possible confounders related to outcome: age, sex, APACHE II score at study entry, SOFA score at study entry, platelet count on day 0, CRP level on day 0 and administration of rhTM. Consequently, three prognostic variables were selected: sex, APACHE II score and administration of rhTM. After adjusting for APACHE II score and sex, rhTM administration was the only parameter identified as an independent significant predictor of the probability of 28-day mortality (adjusted hazard ratio, 0.303; 95% confidence interval, 0.106 to 0.871; *P *= 0.027) (Table 3). The survival curves of the prediction model calculated by Cox regression analysis are shown in Figure 2.

**Table 3:** Independent variables in final multiple regression models by Cox regression analysis^a^

Variables	Coefficient	Hazard ratio	95% CI	*P *value
rhTM administration	-1.193	0.303	0.106 to 0.871	0.027
APACHE II score	0.064	1.066	1.000 to 1.138	0.052
Males	-0.670	0.512	0.235 to 1.115	0.092

^a^rhTM, recombinant human soluble thrombomodulin; APACHE II, Acute Physiology and Chronic Health Evaluation II; CI, confidence interval.

### Effect of treatment on organ damage

**Figure 3:**
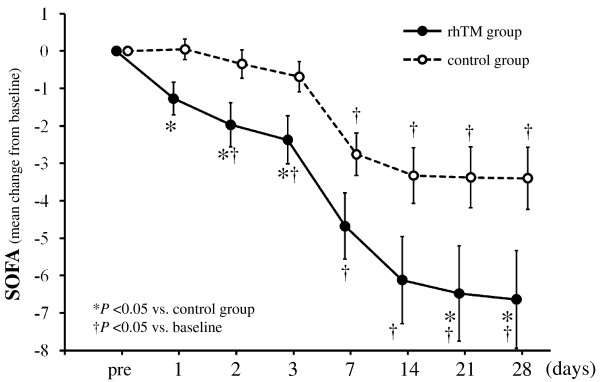
**Serial changes from baseline in SOFA score in the two groups**. Data are expressed as group means ± standard error of the mean. SOFA score decreased over time in both groups (*P *= 0.016). The degree of decrease in SOFA score was significantly greater in the rhTM group than in the control group (*P *= 0.028). There was no interaction between treatment and time (*P *= 0.192). The last-observation-carried-forward method of imputation was used for missing data. The imputation was accomplished in a total of 13 values in four patients for the rhTM group and in a total of 83 values in 27 patients for the control group. *P < 0.05 compared to the control group. †*P *< 0.05 compared to baseline. SOFA, Sequential Organ Failure Assessment; rhTM, recombinant human soluble thrombomodulin.

The serial changes in SOFA score in the two groups are shown in Figure 3. There was a significant difference in the change of SOFA score from baseline to day 28 between the two groups (*P *= 0.028). In the *post hoc *test, the SOFA score rapidly decreased on day 1 in the rhTM group as compared to the control group (*P *< 0.05), and a significant difference between the two groups continued to day 3.

The effect of rhTM on SOFA score was investigated in detail to gain insight into the mechanisms through which this novel biological agent produced a mortality benefit. We evaluated SOFA scores for respiratory, cardiovascular, renal and hepatic organ systems between the two groups from day 0 through day 28. The patients in the rhTM group showed a tendency toward a decrease in respiratory score (*P *= 0.075) and renal score (*P *= 0.069) compared to those in the control group by repeated measures ANOVA, but the differences between the two groups were not statistically significant. No significant differences in cardiovascular score (*P *= 0.190) and hepatic score (*P *= 0.586) were observed between the two groups.

### Effect of treatment on inflammation and coagulation data

**Figure 4:**
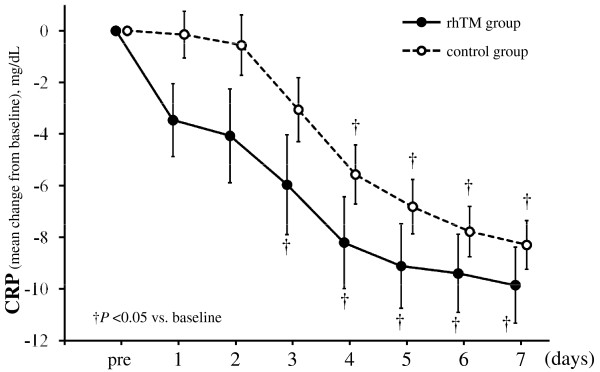
**Serial changes from baseline in levels of CRP in the two groups**. Data are expressed as group means ± standard error of the mean. CRP level decreased over time in both groups (*P *< 0.001). Although CRP level in the rhTM group tended to decrease more than that in the control group, the decrease did not reach statistical significance (*P *= 0.144). There was no interaction between treatment and time (*P *= 0.812). The last-observation-carried-forward method of imputation was used for missing data. The imputation was accomplished in a total of six values in one patient for the rhTM group and in a total of 51 values in 12 patients for the control group. †*P *< 0.05 compared to baseline. CRP, C-reactive protein; rhTM, recombinant human soluble thrombomodulin.

**Figure 5:**
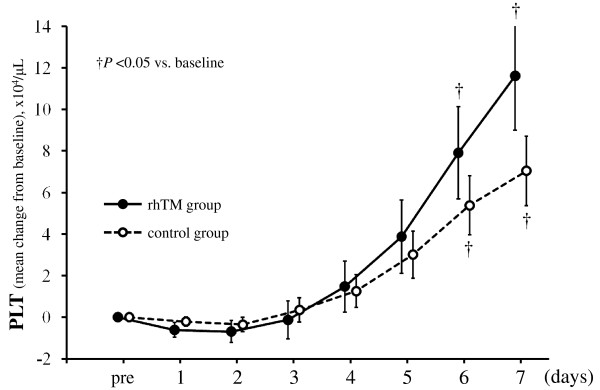
**Serial changes from baseline in platelet counts in the two groups**. Data are expressed as group means ± standard error of the mean. Platelet counts increased over time in both groups (*P *< 0.001). Although the changes in platelet counts between the two groups were not significant (*P *= 0.509), the interaction between treatment and time was statistically significant (*P *= 0.040). The last-observation-carried-forward method of imputation was used for missing data. The imputation was accomplished in a total of six values in one patient for the rhTM group and in a total of 51 values in 12 patients for the control group. †*P *< 0.05 compared to baseline. PLT, platelets; rhTM, recombinant human soluble thrombomodulin.

The serial changes in CRP levels in the two groups are shown in Figure 4. CRP level decreased more quickly in the rhTM group than in the control group, although the difference between the two groups was not statistically significant (*P *= 0.144). There was no difference in the recovery of platelet counts between the two groups (*P *= 0.509). However, the interaction between the treatment and time was statistically significant (*P *= 0.040), suggesting that the recovery of platelet counts in the rhTM group might have been greater than that in the control group after day 5 as shown in Figure 5.

**Figure 6:**
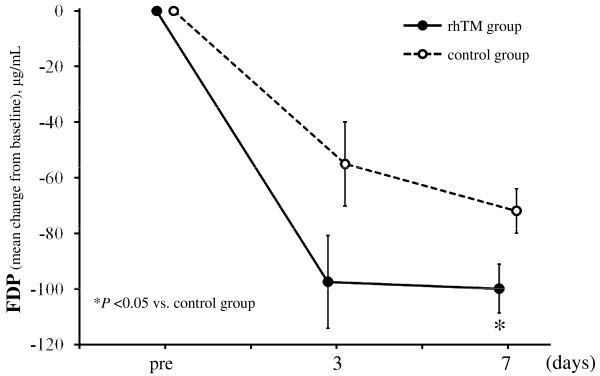
**Serial changes from baseline in levels of FDP in the two groups**. Data are expressed as group means ± standard error of the mean. The degree of decrease in FDP level was significantly greater in the rhTM group than in the control group (*P *= 0.017). The decrease of FDP level over time and the interaction between treatment and time were not significant (*P *= 0.844 and *P *= 0.525, respectively), probably because of small sample size and considerable variation. Because we included in our analysis only patients for whom baseline values and subsequent values were recorded, only 23 of 45 patients were included in the control group. The last-observation-carried-forward method of imputation was used for missing data. The imputation was accomplished in a total of one value in one patient for the rhTM group and in a total of 12 values in 12 patients for the control group. *P < 0.05 compared to the control group. FDP, fibrinogen degradation products; rhTM, recombinant human soluble thrombomodulin.

In terms of FDP analysis, we included only patients for whom baseline values and subsequent values were recorded; thus, FDP data for all patients in the rhTM group and for 23 of 45 patients in the control group were analyzed. The baseline characteristics of the 23 control group patients were not different from those of the other control group patients. There was a significant difference in the change of FDP level from baseline between the two groups (*P *= 0.017) as shown in Figure 6.

### Adverse events

During the study period, one serious adverse event related to bleeding occurred in the rhTM group (5.0%), and two adverse events occurred in the control group (4.4%); however, there was no significant difference in the incidence of this adverse event between groups. The bleeding event in the rhTM group was cerebral hemorrhage requiring craniotomy, from which the patient recovered. The cerebral hemorrhage occurred on day 10, five days after the end of rhTM administration. The cause-and-effect relationship between administration of rhTM and hemorrhage was unclear. The two adverse events in the control group were gastrointestinal bleeding and respiratory tract bleeding, with both patients requiring massive blood transfusion.

## Discussion

The results of this study provide evidence that rhTM may have a beneficial effect on organ dysfunction in patients with sepsis-induced DIC. We have demonstrated a significant decrease in SOFA score in the rhTM group compared to the control group. In addition, Cox regression analysis indicated that 28-day mortality was significantly lower in the patients treated with rhTM than in control patients treated without rhTM.

A few clinical investigations on the effects of rhTM have been reported. Saito *et al*. [15] showed the results of a multicenter, randomized controlled trial that examined the effects of rhTM on DIC patients. However, there were several issues in that trial. First, there was no limitation as to the underlying disease causing the DIC in the study patients. Accordingly, DIC resulted from a hematologic malignancy in half of the patients and from sepsis in the other half. Second, the control group was treated not with a placebo, but with unfractionated heparin. Third, the primary end point was defined as DIC resolution rate, not mortality. Although these investigators demonstrated a significant improvement in the rate of DIC resolution in the rhTM group compared with the heparin group, the effects of rhTM on mortality were not significantly different in the patients with sepsis. There has been no other report in which the effects of rhTM on mortality in patients with sepsis-induced DIC were assessed.

Several animal studies have demonstrated a reduction in mortality in a severe sepsis model with the administration of rhTM. Nagato *et al*. [17] reported that rhTM inhibits the production of inflammatory cytokines, decreases plasma HMGB1 levels and reduces mortality in experimental endotoxemia in rats. Iba *et al*. [18] showed that the changes in coagulation abnormalities were reduced and that mortality was decreased by the concomitant administration of rhTM and AT in rats in which sepsis was induced by lipopolysaccharide infusion. These results suggest that rhTM plays a central role in regulating not only coagulation but also inflammation in sepsis, but the clinical mechanism of action responsible for these effects of rhTM was not fully elucidated. Two different mechanisms have been described for the anticoagulative effects of rhTM [10,19]. One is a pathway through the production of APC, and the other is by direct binding of rhTM to thrombin to disrupt it, thus producing an anti-thrombin effect. TM is a transmembrane protein on the endothelial cell surface, and the thrombin-TM complex activates protein C to produce APC. In the presence of protein S, APC inactivates factors VIIIa and Va, thereby inhibiting further thrombin formation. In addition, TM has the effect of directly combining with thrombin and resolving it. However, the strong anti-thrombin effect of rhTM through this latter mechanism cannot be expected from the usual clinical dosage.

Several mechanisms have been demonstrated for the anti-inflammatory effects of rhTM. The major mechanism is a pathway through the production of APC as mentioned above. In *in vitro *studies, APC has been shown to exert an anti-inflammatory effect by inhibiting the production of inflammatory cytokines (tumor necrosis factor α, interleukin (IL) 1 and IL-6) by monocytes, suppressing the production of NF-κB and limiting the rolling of monocytes and neutrophils on injured endothelium by binding selectins [20]. Recently, APC has been reported to have a strong cytoprotective effect by cleaving toxic extracellular histones produced in sepsis [21]. These anti-inflammatory effects of APC can be expected with the administration of rhTM. Another mechanism is an anti-inflammatory effect that the N-terminal lectin-like domain of rhTM exhibits, which is to sequester and cleave HMGB1, which is released from necrotic cells and modulates several signals that induce a proinflammatory response leading to severe cell damage [13]. Furthermore, it has been reported that the lectin-like domain of rhTM is capable of specific binding to lipopolysaccharide and reduces lipopolysaccharide-induced inflammation [14]. These anticoagulative and anti-inflammatory effects of rhTM can be beneficial in the treatment of sepsis-induced DIC.

Our results showed that there was a significant difference in the serial change of SOFA scores between the rhTM group and the control group. SOFA scores rapidly decreased in the rhTM group compared to the control group even from day 1, in the early period after rhTM administration. A detailed investigation of the effect of rhTM on SOFA score demonstrated that there was a tendency toward a decrease in serial changes in SOFA score for respiratory and renal organ systems between the two groups. These results suggest that rhTM may have an early beneficial effect on multiple organ damage resulting from severe sepsis. Vincent *et al*. [22] showed in subgroup analysis of the PROWESS trial that patients in the rhAPC group had significantly decreased SOFA scores for cardiovascular and respiratory dysfunction (*P *= 0.009 for both) compared to the control group for days 1 to 7. The beneficial effects of rhTM on organ damage in the present study were similar to those of rhAPC. Procoagulant activity in the setting of acute lung injury and acute respiratory distress syndrome has been recognized. Various animal studies have shown a consistent reduction in lung injury with the administration of anticoagulants such as tissue factor pathway inhibitor, AT and rhAPC [23]. Uchiba *et al*. [24] showed that rhTM prevents endotoxin-induced pulmonary vascular injury in rats by inhibiting pulmonary accumulation of leukocytes through thrombin binding and subsequent protein C activation. These results indicate that rhTM may have a protective effect on lung injury induced by sepsis.

In this study, we have demonstrated a significant decrease in FDP in the rhTM group compared to the control group. Saito *et al*. [15] showed that the rate of change in D-dimer in the rhTM group was significantly higher than that in the heparin group, suggesting that rhTM is superior to heparin in the attenuation of the hypercoagulable state in the phase III trial of rhTM for DIC patients in Japan. In the PROWESS trial, plasma D-dimer levels were significantly lower in the rhAPC group than in the control group on day 1 after the start of infusion [5]. These results indicate that rhTM can improve the hypercoagulative state of sepsis-induced DIC at an early stage. Because microvascular dysfunction may be the key to the development of multiple organ failure in severe sepsis, the microcirculation should be a principal therapeutic target. Suppressing the hypercoagulative state by rhTM administration at early onset of severe sepsis may potentially prevent the progression to multiple organ failure.

In regard to the anti-inflammatory effects of rhTM, although CRP level tended to decrease more quickly in the rhTM group than in the control group, the difference between the two groups was not statistically significant because of the small sample size of the present study. Dhainaut *et al*. [25] showed in subgroup analysis of the PROWESS trial that IL-6 levels fell more rapidly in the rhAPC group than in the control group. Although we did not evaluate cytokine levels in our two groups, a similar inhibitory effect on proinflammatory cytokine production may be expected with the use of rhTM. Further clinical investigation is necessary to clarify the anti-inflammatory activities of rhTM.

Cox regression analysis indicated that 28-day mortality of the patients treated with rhTM was significantly improved in comparison to that in the patients treated without rhTM. We used multivariate analysis in our study because of the significant difference in the severity of illness at baseline between the two groups. Because all patients in the two groups were selected using the same eligibility criteria, the reason for the difference in severity between the two groups was not clear. We extracted candidate prognostic variables possibly related to outcome in the performance of Cox regression analysis. As a result, rhTM administration was revealed to be an independent predictor of probability of 28-day survival. The effects of rhTM on mortality in patients with sepsis-induced DIC require further elucidation.

Bleeding was the most significant adverse event associated with the administration of rhTM, as it is with rhAPC [26]. rhTM is considered to have some favorable effects on the reduction of bleeding complications as compared with rhAPC. First, rhTM has been shown to have a wider safety margin and to have a favorable antithrombotic profile with less bleeding in animals and in *in vitro *experiments [19]. Second, the anticoagulative effect of rhTM depends on the amount of thrombin available. Accordingly, after controlling thrombin generation by rhTM administration, rhTM does not work in excess and generation of further rhAPC decreases. Although the clinical data on rhTM are limited, rhTM appears to result in fewer bleeding complications than rhAPC. In the phase III trial of rhTM in Japan, the incidence of bleeding complications was lower in the rhTM group than in the heparin group (*P *= 0.0487) [15]. In the present study, there was no increase of adverse events related to bleeding in the rhTM group compared with the control group. In the one patient in the rhTM group with cerebral hemorrhage, the cause-and-effect relationship between administration of rhTM and hemorrhage was not clear. Because of the small sample size of this study, future investigation into bleeding complications of patients treated with rhTM is required.

We acknowledge several limitations of our observational study design. First, this study was not a randomized controlled trial, and we compared the rhTM treatment group with a historical control group. Multiple unmeasured variables might account for the outcome differences observed in this study. Second, a small number of patients were included in this study. Third, this study was carried out in a single institution. Further multicenter, prospective, randomized trials are needed to thoroughly evaluate the effects of rhTM on the treatment of sepsis-induced DIC.

## Conclusions

In conclusion, we found that rhTM administration may improve organ dysfunction in patients with sepsis-induced DIC, as demonstrated by the significant reduction in SOFA score. Further clinical investigations are necessary to evaluate the effect of rhTM on the pathophysiology of sepsis-induced DIC.

## Key messages

• rhTM administration may improve organ dysfunction due to severe sepsis as demonstrated by the significant reduction in SOFA score.

• Additional well-designed intervention studies are urgently needed to prove the clinical effectiveness and safety of rhTM.

## Abbreviations

ANOVA: analysis of variance; APACHE: Acute Physiology and Chronic Health Evaluation; APC: activated protein C; AT: antithrombin; CRP: C-reactive protein; DIC: disseminated intravascular coagulation; FDP: fibrinogen degradation products; HMGB1: high-mobility group box 1 protein; ICU: intensive care unit; IL: interleukin; LOCF: last observation carried forward; PROWESS: Recombinant Human Activated Protein C Worldwide Evaluation in Severe Sepsis; rhAPC: recombinant human activated protein C; rhTM: recombinant human soluble thrombomodulin; SOFA: Sequential Organ Failure Assessment; TM: thrombomodulin.

## Competing interests

The authors declare that they have no competing interests.

## Authors' contributions

KY participated in study design and data collection and interpretation, performed the statistical analysis and drafted the manuscript. SF conceived the study and its design and helped to draft the manuscript. TM, HM and YN participated in study design and data collection. THirose, OT, YK and TS participated in data interpretation. HO had a major impact on the interpretation of data and critical appraisal of the manuscript. THamasaki performed the statistical analysis and helped to draft the manuscript. All authors read and approved the final manuscript.
